# The Practice of Surgery

**Published:** 1846-04

**Authors:** 


### Art. XV.

*The Practice of Surgery.* By James Miller, f.r.s.e., Professor of Surgery in the University of Edinburgh, Surgeon to the Royal Infirmary, &c. &c.—*Edinburgh,* 1846. 8vo. pp. 680.

We had occasion in a former Number of this Journal (April, 1845) to speak in deservedly high terms of Professor Miller's work on the Principles of Surgery, and we are happy to be able to pronounce an equally favorable judgment on the manner in which the present volume is executed.

The same reason which prevented our undertaking to give anything like a complete review of the preceding work, namely the extent of the field embraced by it, must be our plea for contenting ourselves with selecting portions here and there, as specimens of the manner in which the author has performed his task, rather than attempting a condensed survey of the whole.

The author has chosen the division of the body into regions as the groundwork of his arrangement, commencing with the injuries and diseases to which the head is liable, and passing successively to those of other parts of the body. This order is perhaps the most convenient. The objections that may fairly be made to it, viz. that analogous affections, such, for instance, as fractures and dislocations of the upper and lower extremities, are unduly separated from each other, will have less weight when we remember that the general principles of treatment have formed the subject of a preceding volume, and that the practical application of those general principles is alone the object of the present.

The chapters devoted to injuries of the head and brain, and to diseases of the eye, furnish a good epitome of the present state of practice in these important parts of surgery; that on diseases of the scalp is derived chiefly from Mr. Erichsen's excellent treatise. The chapter on affections of the neck is carefully and judiciously written. The following are the author's conclusions respecting the diseases in which bronchotomy or tracheotomy should be employed:

"*Bronchotomy*, then, is available in the following cases: 1. In the case of *foreign bodies* lodged in the air passages. Extrusion, independently of this operation, is and ought to be the exception to the general rule. 2. In *suspended animation;* when we cannot otherwise effect with certainty artificial inflation of the lungs. 3. In *spasm of the glottis.* Threatened asphyxia from external injury may depend on this cause—perhaps on a precisely opposite condition; in either case the operation is imperatively demanded to save life. There is the same necessity in the spasmodic occlusion of the glottis which attends poisoning by carbonic acid. In *laryngismus stridulus* we withhold the operation if possible, and trust to general treatment; yet we are aware that urgent circumstances may arise to demand the tracheal wound, at least with the hope of palliation, and perhaps with the hope of affording time for the effectual working of other remedies. 4. In *œdema glottidis,* chronic or acute, there is no safety but by operation, as soon as the symptoms have become at all urgent. And in the acute cases there is the best hope of speedy discontinuance of the tube, closure of the aperture, and complete restoration of normal respiration. 5. In *laryngitis fibrinosa,* the operation is as warrantable as in urgent œdema, when the disease is limited to the larynx. But in the great majority of cases of true croup, in which the whole windpipe with its ramifications is involved, operation is surely to be regarded as a rare exception to the general rule of non-interference; in the early stage it is inexpedient, while mechanical obstruction to respiration is not yet threatened; in the more advanced period it is ineffectual—failing to fulfil the objects of its performance. 6. In *purulent laryngitis* there is the same necessity for operation and the same good prospect as to its result as in acute œdema. 7. In *chronic laryngitis*, with thickening, supervention of œdema, through inflammatory accession, may render the operation indispensable to the preservation of life. 8. In *simple ulceration* the same event may occur as that just mentioned in connexion with mere thickening of the membrane. Or, independently of the occurrence of such an accidental crisis, the operation may be deemed expedient, to assist the action of other remedial means and by effecting early cicatrization to save structure and function. 9. In *ulceration and disease of the cartilage* operation is likely to be required to save life from threatened asphyxia, but with little or no prospect of discontinuance of the tube's use. 10. In *phthisis laryngea*, it may be similarly demanded for a temporary object, scarcely with a hope of contributing to a cure, but rather as a means of protraction and palliation. 11. In *pressure on the windpipe*, caused by the formation of a tumour or abscess, or by impaction of food or a foreign body in the œsophagus or pharynx, operation may be necessary if the obstruction to respiration cannot be otherwise relieved—namely, by removal of the cause, by evacuation of the matter, extirpation or diminution of the tumour, or extension of the impacted substance. 12. In *cut throat*, tracheotomy is not unfrequently demanded, imperiously, to save life from impending asphyxia, and it may be expedient, at an early period of the case, to avert all such hazard, and to favour as well as permit entire closure of the wound. 13. In *glossitis*, in *tonsillitis*, and in extreme cases of *pharyngitis*, it is required when swelling is so great, rapid, and uncontrollable as otherwise to render fatal asphyxia all but inevitable. 14. In *carotid aneurism* of large size, when by circumstances we are precluded from speedy recourse to deligation of the artery, life may be suddenly brought into peril by supervention of the diffuse form on the circumscribed, and consequent compression of the windpipe. Bronchotomy then is essential; and the tube will require to be worn until by deligation of the artery we have effected such diminution of the bulk of the tumour as altogether to free the respiratory canal. *Thoracic aneurisms*, be it remembered, by compressing the air passages, may stimulate the results of inflammatory disease in the larynx; and in such cases no good can be expected to result from bronchotomy.

"In the great majority of instances tracheotomy is preferable to laryngotomy for obvious reasons." (p. 270.)

From the respiratory we must make a rapid descent to the urino-genital organs, passing by the intermediate parts and their diseases. On the subject of calculous affections we have a chapter occupying about sixty pages, the first nine of which only are allotted to the description and treatment of urinary deposits. This we consider a somewhat meagre allowance for so important a part of pathology, in which the surgeon is equally interested with the physician; and we think a chapter on the principles might with advantage have been dedicated to the subject. The mode of operating recommended by the author in lithotrity is that of Mr. Liston, the greatest authority in the present day on this subject. The following are the directions given for avoiding urinary infiltration, the most serious and frequent of the evil attendants on lithotomy; these, though not new, cannot be too carefully borne in mind:

"To obviate it the following points are of essential importance: Maintain the reflexion of the ileo-vesical fascia entire, at the base of the prostate, that gland being not divided throughout its whole extent by the knife, but rather first notched, and then dilated by the finger and forceps. Make the general wound conical in form, the base at the integument of the perineum the truncated apex at the prostate. Make the general wound also sloping in form, its fall being from the prostate obliquely downwards—cutting obliquely up to the bladder, not directly into it; also arranging the patient's trunk in bed so as to favour this sloping form, obviously so well calculated for the ready draining away of the urine. In using the finger in dilatation avoid all laceration, torn parts being but ill disposed for rapid plastic exudation. Retain the tube for the necessary number of hours, and keep it clear from coagulum or other source of obstruction. I have latterly thought it advisable to use a somewhat larger tube than that in general use, in the belief that it is better adapted for preventing urinary infiltration; as, while it affords a more ready exit for that fluid, it compresses the track of the deep wound, and may be supposed to afford a very effectual barrier to entrance of urine into the cellular tissue. Further experience of its use is, however, necessary, to determine whether or not it may do harm by delaying the closure of the wound and exciting inflammation at the neck of the bladder. Further, the risk by infiltration is certainly diminished by not operating unless the urinary organs and general system are free from excitement, the kidney acting healthily, and the urine in a satisfactory condition; and also by maintaining after the operation a supply of urine which is bland as well as copious, mainly aqueous, and containing but a sparing amount of saline matter. For if infiltration do occur to some extent, it will be less hazardous to part and system under such circumstances than if the infiltrated urine were the scanty and acrid urine of fever or of renal disease." (p. 460.)

On the subject of the treatment of aneurism by pressure on the artery above the tumour, the author has the following observations:

"Popliteal aneurism is probably the most common of all external aneurisms; and hitherto the Hunterian application of ligature to the superficial femoral has been the only approved mode of treatment. Latterly, however, as formerly explained, (Principles, p. 457,) the application of pressure instead of the ligature has been proposed. And experience is almost daily giving direct and undoubted testimony to the efficacy of the practice. There are some patients, doubtless, who may prove intolerant of pressure, and there may be others who prefer the apparent certainty of the knife and ligature to the apparent uncertainty and delay of the compressor; but the vast majority of cases are assuredly capable of cure by pressure properly applied, without risk, with but little pain or inconvenience, and without any wearisome amount of privation or confinement. The skin which is to bear the pressure is protected by a layer of thick soap plaister, and that again may be covered by leather. And more than one compressor is used, or at least pressure is made at different parts at different times; so that the burthen of it may not all be thrown on one point, but by being subdivided among several points may be rendered much more tolerable. Using several instruments along the course of the vessel in the thigh, they may be slackened and tightened alternately; or the same instrument may be shifted in its site with a like effect. It is never to be forgotten that all severity of pressure is unnecessary—that it is not essential that it should be such as to arrest the arterial flow at the compressed point—that, on the contrary, consolidation of the artery is more likely to take place when a slow, and gentle, and feeding circulation remains. And it is also important to remember that should this mode of treatment fail, it by no means interferes with the subsequent performance of the ordinary operation, but, on the contrary, renders its success all the more probable." (p. 609.)

We had occasion to remark on certain little peculiarities of style in the former volume, which we thought might with advantage be altered. These, if they have not wholly disappeared in the present, appear, we are bound to say, in a very mitigated form; and we feel no hesitation in recommending Professor Miller's two volumes as affording to the student what the author intended, namely, a complete Text Book of Surgery. Taken altogether, indeed, the work is one of a highly creditable kind, and full of such proofs both of talent and knowledge, as justify us in expecting something of yet higher mark and likelihood from its accomplished author.

---

